# Performance of Epigenetic Markers SEPT9 and ALX4 in Plasma for Detection of Colorectal Precancerous Lesions

**DOI:** 10.1371/journal.pone.0009061

**Published:** 2010-02-04

**Authors:** Marc Tänzer, Benjamin Balluff, Jürgen Distler, Kari Hale, Andreas Leodolter, Christoph Röcken, Bela Molnar, Roland Schmid, Catherine Lofton-Day, Tibor Schuster, Matthias P. A. Ebert

**Affiliations:** 1 Department of Medicine II, Klinikum rechts der Isar, Technische Universität, München, Germany; 2 Epigenomics Inc., Seattle, Washington, United States of America; 3 Department of Medicine, Jung-Stilling Krankenhaus, Siegen, Germany; 4 Institute of Pathology, Christian-Albrechts-University, Kiel, Germany; 5 Department of Medicine II, Semmelweis University, Budapest, Hungary; 6 Institute of Medical Statistics and Epidemiology, Klinikum rechts der Isar, Technische Universität, München, Germany; The University of Hong Kong, Hong Kong

## Abstract

**Background:**

Screening for colorectal cancer (CRC) has shown to reduce cancer-related mortality, however, acceptance and compliance to current programmes are poor. Developing new, more acceptable non-invasive tests for the detection of cancerous and precancerous colorectal lesions would not only allow preselection of individuals for colonoscopy, but may also prevent cancer by removal of precancerous lesions.

**Methods:**

Plasma from 128 individuals (cohort I – exploratory study: 73 cases / 55 controls ) was used to test the performance of a single marker, *SEPT9*, using a real-time quantitative PCR assay. To validate performance of SEPT9, plasma of 76 individuals (cohort II – validation study: 54 cases / 22 controls) was assessed. Additionally, improvement of predictive capability considering SEPT9 and additionally ALX4 methylation was investigated within these patients.

**Results:**

In both cohorts combined, methylation of SEPT9 was observed in 9% of controls (3/33), 29% of patients with colorectal precancerous lesions (27/94) and 73% of colorectal cancer patients (24/33). The presence of both SEPT9 and ALX4 markers was analysed in cohort II and was observed in 5% of controls (1/22) and 37% of patients with polyps (18/49). Interestingly, also 3/5 (60%) patients with colorectal cancer were tested positive by the two marker panel in plasma.

**Conclusions:**

While these data confirm the detection rate of *SEPT9* as a biomarker for colorectal cancer, they also show that methylated DNA from advanced precancerous colorectal lesions can be detected using a panel of two DNA methylation markers, *ALX4* and *SEPT9*. If confirmed in larger studies these data indicate that screening for colorectal precancerous lesions with a blood-based test may be as feasible as screening for invasive cancer.

## Introduction

Colorectal cancer (CRC) is the second most frequent cancer in Europe and the US affecting 412,900 and 150,000 individuals in 2006, respectively [1], [2]. CRC is also one of the leading causes of cancer deaths in western countries, accounting for 207,400 deaths in Europe in 2006 alone [2]. Interestingly, the number of deaths from CRC has increased in Europe by 1.8% since 2004, despite recent improvements in its diagnosis and therapy. While screening has been shown to be effective in terms of reduction of disease-related mortality and costs [3], [4], the identification of early stages of CRC or even precancerous lesions is hampered by the low performance of non-invasive screening tests and the low acceptance and compliance of invasive screening using colonoscopy.

A novel approach of molecular testing for CRC is the analysis of DNA in stool. In a study published by Imperiale et al. [5], fecal DNA testing was superior to FOBT in the detection of invasive cancers and adenomas with high-grade dysplasia. Fecal DNA testing was positive in 18% of individuals confirmed to have advanced neoplasia (CRC or large adenomatous polyps) while FOBT was positive in only 10.8%. Overall, fecal DNA studies report sensitivities from 62 to 91% for CRC compared to 15–80% for FOBT [6], [7].

Evidence is increasing that, apart from genetic alterations, epigenetic changes are of similar importance in the pathogenesis of CRC. Particularly tumor suppressor genes can be silenced by the methylation of CpG islands, which are found in the 5′ region of approximately half of all human genes [8], [9]. Several groups have demonstrated a high frequency of somatic gene methylation in CRC, and transcriptional silencing in the tumor cells has been reported for an increasing number of genes, including *p16INK4, MGMT, GSTP1, CDH1, APC* and *TIMP3* among many others [10], [11]. Besides, these epigenetic alterations occur already in the early stages of tumor development, including precancerous lesions, such as adenomas, which indicates that the analysis of epigenetic DNA alterations may be useful for the diagnosis of malignant diseases. Previously we independently detected a significant higher prevalence of methylated *ALX4* and *SEPT9* DNA in peripheral blood of colorectal cancer patients compared to controls [12], [13]. Using *SEPT9* methylated DNA, CRCs were predicted with a sensitivity of 72% and a specificity of 93% [13]. Moreover, in one study, large polyps were detected in approximately 20% of patients using the analysis of *SEPT9* methylation in peripheral blood [14]. Based on these previous reports on the potential use of *SEPT9* for the detection of early precancerous lesions we decided to further evaluate this marker in patients with polyps. Therefore, we designed this study, including an exploratory and a validation study, in order to further assess the performance of *SEPT9* methylated DNA as a marker in the detection of colorectal precancerous lesions. In the validation study, we included also methylated *ALX4* DNA to test if the predictive value for polyps increases if an additional marker is used, this has to be further validated, however. Since early non-invasive detection of polyps could be followed by endoscopic removal of precancerous lesions, this strategy might help to prevent more invasive colorectal cancers.

## Methods

### Subjects for Methylation Analysis

The entire study population was assembled by two seperate cohorts. For cohort I (exploratory cohort), plasma was collected from 128 individuals with different clinical characteristics: healthy controls (n = 12), patients with symptomatic non-malignant bowel diseases with or without positive FOBT (n = 17), patients with a history of polypectomy or family history for colorectal neoplasia (n = 6), patients with chronic inflammatory diseases of the gastrointestinal tract (n = 20), 45 patients with colorectal precancerous lesions and 28 patients with CRC (Table 1).

**Table 1:** List of patients and clinical data for cohort I (exploratory) and cohort II (validation).

Cohort I (exploratory)					
	Total Samples	Male/Female	Median Age	Age Range	Disease
Normal	12	5 / 7	60.5	52–72	healthy, no symptoms
Polyp	45	2 / 835 not stated	64(10 patients)	44–71	35 unclassified adenomas5 tubular adenomas1 serrated adenoma3 high grade invasive tubular adenomas1 high grade invasive tubulo-villous
CRC	28	2 / 224 not stated	67(4 patients)	63–71	5 stage 02 stage I14 stage IV7 unknown stage
Inflammation	20	2 / 711 not stated	61(9 patients)	31–80	9 colitis or gastritis1 anastomositis1 ulcus1 collagene colitis1 M. Crohn1 NSAID-colopathy with positive FOBT1 antibiotics1 diverticulitis2 recurring diarrhea1 diarrhea and positive FOBT1 diarrhea and bowel pain
Risk patient	6	3 / 3	47.5	39–68	2 after polpectomy2 familial history2 M2-PK positive
Symptomatic	17	9 / 8	57	22–84	6 positive FOBT3 irregular stool2 constipation2 bleedings1 loosing weight1 flatulence2 bowel pains
	128				
Cohort II (validation)					
	Total Samples	Male/Female	Median Age	Age Range	Disease
Normal	22	11 / 11	42.5	25–69	
Polyp	49	31 / 18	63	20–75	36 adenomas, 13 hyperplastic polyps
CRC	5	3 / 2	59	27–79	4 stage I1 stage III
Total	76				

For cohort II (validation cohort), plasma samples were collected from 76 individuals prior to any intervention: 49 patients (18 female, 31 male) with colorectal precancerous lesions (36 with adenomas, 13 with hyperplastic polyps; patients' median age 63 years, range 20–75 years), from 22 healthy controls (11 female, 11 male; median age 42.5 years, range 25–69 years) without colorectal lesions and 5 patients (2 female, 3 male) with CRCs (median age 59 years, range 27–79 years; 4 patients UICC stage I, 1 patient UICC stage III, Table 1).

Biopsies were taken during resection and/or endoscopy and were formalin fixed [15]. Sections were stained with hematoxylin and eosin for histological evaluation [15]. Five randomly selected CRC patients were included in this analysis and served as positive controls. In all cases tumor stages were assessed using the TNM-system according to the guidelines of the Union Internationale Contre Le Cancer (UICC) [16]. This study was approved by the Ethics Committee of the Technical University Munich, Germany. Written consent from all patients was obtained.

### DNA Extraction

Genomic DNA was either extracted from 12 ml plasma using a MagnaPure® device (Roche) and the MagNAPure LC Total Nucleic Acids Large Volume Extraction Kit (Roche Diagnostics #03264793001) as previously reported [12] or was extracted and bisulfite converted using the SEPT9 Detection Assay (Epigenomics #M4-01-003). SEPT9 methylation analysis was done by Heavy MethyLight as described below. The assay workflow yielded 2–5 µg/l circulating plasma DNA following bisulfite treatment. Recovery was, thus, 45–50% of genomic DNA, similar to previous reports (Figures S1 and S2) [13].

### SEPT9 Methylation Analysis by Heavy MethyLight

SEPT9 methylation was determined using the HeavyMethyl technique [17]. In the HeavyMethyl technique four oligonucleotides are used per reaction including two non-methylation specific PCR primers, one with an overlapping blocker sequence specific for unmethylated DNA, flanking a methylation specific probe. The forward primer for SEPT9 was 5′-GTAGTAGTTAGTTTAGTATTTATTTT-3′, the reverse primer was 5′-CCCACCAACCATCATAT-3′. Probe sequences were GTTCGAAATGATTTTATTTAGTTGC-FL and LC-Red640-CGTTGATCGCGG GGTTC-PH. The blocker sequence was 5′-CATCATATCAAACCCCACAA TCAACACACAAC-3′. A C3 spacer was introduced at the 3′ end of this sequence.

### ALX4 Methylation Analysis by Quantitative Real-Time PCR

Genomic DNA was analysed by the MethyLight technique after bisulfite conversion as previously reported [18], [19]. In the MethyLight analysis three oligonucleotides are used in every reaction. Two locus-specific PCR primers flank an oligonucleotide probe with a 5′ fluorescent reporter dye (6FAM) and a 3′ quencher dye (BHQ-1). For this analysis primers and probes are specifically designed to bind to bisulfite-converted DNA, which generally span 7 to 10 CpG dinucleotides. The gene of interest is then amplified in case of complete methylation. The specificity of the reactions for methylated DNA is confirmed using human sperm DNA (unmethylated) and CpGenome Universal Methylated DNA (Chemicon (subsidiary of Serologicals) catalog #S7821) (methylated). The primer and probe sequences were sALX4f, 5′-CGTCGCAACGCGTACG-3′, sALX4r, 5′-CGCGGTTTCGATTTTAATGC-3′. Probe sequences were 5′-ACTCCGACTTAACCCGACGATCG-3′ and 5′-ACGAAATTCCTA ACGCAACCGCT-3′ [12]. No sequencing was performed and no new data added to Genbank.

### Statistical Analysis

The results of the MethyLight and HeavyMethyl assays were interpreted in a purely qualitative way as previously reported [14]. We confirmed that the sensitivity of the MethyLight and HeavyMethyl assays are equivalent in previous studies [13], [14]. The limit of detection (LOD) was 25 pg for these assays which were performed as previously reported [14] (Supplementary file 2). For the qualitative analysis of two methylation markers, amplification curves above the baseline indicated presence of methylated DNA in the plasma samples. The different clinicopathological features, such as size of lesion and histologic subtype of lesion were used as categorial variables. Frequency distribution of these parameter values were compared using the Fisher's exact test or Chi square test. All tests were two-sided, and a *p-*value of <0.05 was considered statistically significant [18], [19]. To retain a maximum of power in the primary interesting analyses, no correction of alpha error rate in consideration of the multiple test issue was performed. Concerning this matter, we follow a more practical solution: as suggested by Saville [20], corrections for multiple comparisons are not performed but all available data and comparisons made are honestly reported allowing the reader to draw the conclusions. So the reader can informally adjust for multiple comparisons while reviewing the data.

## Results

### Single Marker Analysis in Plasma Samples (Cohort I - Exploratory Cohort)

First, we determined the presence of a single DNA methylation marker in plasma from patients with colorectal precancerous lesions, CRC, in healthy controls without any colorectal pathology, patients with chronic inflammation, or other gastrointestinal diseases and patients with a high risk for CRC due to family history or positive FOBT (Table 2). All methylation marker measurements were performed in triplicate. For the analysis of the marker panel performance two different classification methods were selected. The first method classified patients as positive if both single marker classifications were positive (2 or 3 measurements). The second method classified patients as positive if independent of the marker any three of the total six PCR reactions showed amplification.

**Table 2:** Analysis of *SEPT9* methylation in plasma samples.

cohort I (exploratory)		2/3 positive		1/3 positive	
SEPT 9	Total Samples	Patients (n)	%	Patients (n)	%
Normal-healthy control	12	2	17	3	25
Polyp	45	21	47	28	62
CRC	28	22	79	25	89
Inflammation	20	10	50	18	90
High risk patient	6	4	66	6	100
Symptomatic patient	17	9	53	17	100
cohort II (validation)		2/3 positive		1/3 positive	
Normal-healthy control	22	1	4.5	1	4.5
Polyp	49	6$	12	15*	31
CRC	5	2	40	2	40
both cohorts combined		2/3 positive		1/3 positive	
Normal-healthy control	34	3	9	4	12
Polyp	94	27&	29	43§	46
CRC	33	24	73	27	82

p-values Fisher's exact test (2-sided) polyp vs. normal:

$ 0.43;

* 0.01;

& 0.02 ;

§ 0.0004.

*SEPT9* methylated DNA in 1 of 3 measurements was detected in 3 out of 12 healthy controls (25%), 28 out of 45 patients with polyps (62%), 25 out of 28 (89%) patients with colorectal cancers, 18 out of 20 (90%) patients with chronic inflammation and 6 of 6 high risk patients as well as 17 out of 17 patients with symptomatic diseases. Using the cut-off of at least 2 or 3 positive measurements only 2 of 12 healthy controls (17%), 9 of 17 symptomatic patients (53%), 4 of 6 risk patients (67%), 10 of 20 patients with inflammation (50%), 21 of 45 patients with colorectal precancerous lesions (47%) and 22 of 28 CRC patients (79%) were classified as positive (Table 2).

### Single Marker Analysis in Plasma Samples (Cohort II - Validation Cohort)

Since in the exploratory study positive *SEPT9* levels were found in various non-malignant intestinal diseases, we used a second cohort to assess the performance of *SEPT9* in comparison to asymptomatic healthy controls. *SEPT9* methylated DNA in 1 of 3 measurements was detected in 1 of 22 (5%) healthy controls. In contrast, *SEPT9* methylation was significantly more frequent in patients with polyps (15 of 49 patients (31%); p = 0.01). Using the cut-off of at least 2 or 3 positive measurements with regard to the presence of *SEPT9* methylated DNA in plasma, again only 1 of 22 (5%) healthy controls was classified as positive, whereas patients with polyps exhibited methylated *SEPT9* DNA in at least 2 measurements in 6 of 49 (12%) cases. Cancer patients were positive in 2 of 5 cases using either classification (Table 2).

### Single Marker Analysis in Exploratory + Validation Cohort

Combining the data from both cohorts for methylated *SEPT9* DNA gave the following results: Under the high specificity criteria, 3 out of 34 (9%) healthy controls and 27 out of 94 polyp patients (29%) as well as 24 out of 33 (73%) CRC patients were classified as positive. The difference in prevalence of SEPT9 DNA between polyp and control samples was statistically significant (p = 0.02, Table 2). Using the high sensitivity determination, the positive fraction in the healthy controls raised to 4 out of 34 (12%), whereas 43 out of 94 polyp (46%) and 27 out of 33 (82%) cancer patients were considered positive. Again the difference between polyps and normal controls was statistically significant (p = 0.0004, Table 2).

### Analysis of Marker Panel Performance in Plasma Samples

In order to improve the detection rate of *SEPT9* in patients with colorectal precancerous lesions, we performed a two marker panel analysis in the validation cohort. First we assessed the performance of the single marker *ALX4* in these patients. In the high sensitivity determination, *ALX4* methylation was detectable in 13 of the 22 healthy controls (59%), 38 of the 49 polyp patients (78%) and in 4 of 5 (80%) cancer patients. Using the more stringent criteria (high specificity) the number of healthy controls with positive DNA detection decreased to 4 of 22 individuals (18%), whereas for patients with polyps 22 of 49 patients (45%) were considered positive (p = 0.02). Methylated DNA was observed in 2 of 5 cancer patients (Table 3).

**Table 3:** Analysis of *ALX4* methylation in plasma samples cohort II (validation).

		2/3 positive		1/3 positive	
ALX4	Total Samples	Patients (n)	%	Patients (n)	%
Normal-healthy control	22	4	18	13	59
Polyp	49	22*	45	38$	77.5
CRC	5	2	40	4	80

p-values Fisher's exact test (2-sided) polyp vs. normal:

$ 0.15;

* 0.02

Next, we analysed the two marker panel with regard to performance in the detection of colorectal precancerous lesions. Again, two different levels of stringency with regard to the presence of methylated DNA in plasma samples were applied. Using *SEPT9 + ALX4* and classifying a sample as “positive” if any of the single markers is “positive” (at least 2 of the 3 measurements), we observed 4 positive cases among the 22 healthy controls (18%), 25 positive cases among the 49 patients with polyps (51%) and 3 positive cases among the 5 cancer patients (Table 4). Thus, there was a significantly higher frequency of methylated markers in plasma of patients with polyps versus healthy controls (p = 0.0068).

**Table 4:** Two marker panel analysis in plasma – high specificity criteria and high sensitivity criteria.

		2/3 positive either assay		3/6 positive	
SEPT 9 and ALX4	Total Samples	Patients (n)	%	Patients (n)	%
Normal-healthy control	22	4	18	1	5
Polyp	49	25$	51	18*	37
CRC	5	3	60	3	60

p-values Fisher's exact test (2-sided) polyp vs. normal:

$ 0.0068;

* 0.0013.

Applying the less stringent criteria of at least 3 positive measurements among the 6 combined measurements of both markers, 1 of 22 healthy controls was classified as positive (5%), whereas 18 of 49 patients with polyps (37%) and, again, 3 of 5 cancer patients were found to be positive. The difference between normals and polyps was highly statistically significant (p = 0.0013) (Table 4).

### Marker Performance and Subclassification of Polyps

Next we turned to the analysis of marker performance with respect to the different types of polyps analysed in our study. Polyps were subclassified with respect to the clinically relevant size of <10 mm and ≥10 mm and histomorphological criteria (e.g. tubular or tubulovillous type, presence of high grade or low grade intraepithelial neoplasia). Patients with tubular or tubulo-villous adenomas irrespective of size were positive with regard to the presence of the marker panel in blood in 19 of 36 (53%) patients. The frequency of detectable methylated DNA increased in the subclass of patients with adenomas ≥10 mm (69%, 11 of 16). In the subgroup of polyps ≥10 mm and the presence of high grade intraepithelial neoplasia methylated DNA of the two markers was even observed in the plasma in 5 of 7 cases (71%; Table 5). Overall, methylation markers were significantly more frequent in patients with advanced colorectal adenomas compared to healthy controls (p<0.001). Using a panel of the 2 markers in plasma, sensitivity and specificity for the detection of advanced precancerous colorectal lesions was 71% and 95%, respectively (Tables S1 and S2).

**Table 5:** Analysis of marker performance with regard to polyp histology.

Polyp characteristics	Cases	*SEPT9* 2/3^x^		*ALX4* 2/3		*SEPT9* or *ALX4* 2/3		*SEPT9* + *ALX4* 3/6	
		Patientts (n)	%	Patientts (n)	%	Patientts (n)	%	Patientts (n)	%
> or = 10 mm	18	3	17	9^o^	50	12^x,^ §	67	9§	50
<10 mm	31	3	10	13^o^	42	13*	42	9^o^	29
Hyperplastic	13	1	8	6^x^	46	6^x^	46	4^x^	31
Tubular or tubulovillous adenoma-all sizes	36	5	14	16^o^	44	19^o^	53	14§	39
Tubular or tubulovillous adenoma, >10 mm	16	3	19	8^o^	50	11§	69	8§	50
Tubular or tubulovillous, >10 mm + aIEN	7	1	14	4^x^	57	5^o^	71	5*	71
Normal control	22	1	4.5	4	18	4	18	1	5

Legend: x, polyp versus normal: not significant;

o, lesion versus normal colon: p<0.05;

$, polyp size ≥10 mm versus <10 mm: p<0.05;

§, lesion versus normal colon: p<0.005;

*, lesion versus normal colon: p<0.001; aIEN, advanced intraepithelial neoplasia.

## Discussion

The main problem of CRC screening today is low compliance in existing screening programmes. Despite the fact that CRC has all features which qualify this disease for mass screening, including high incidence and prevalence, high mortality in advanced disease, potential for cure in early stages, on average less than 30% of the eligible population in the US and Europe actually undergo these preventive procedures [3], [4]. Preselection of individuals with precancerous or early neoplastic lesions with a patient-friendly non-invasive molecular test followed by colonoscopy for diagnosis and potential removal of these lesions, may not only improve compliance and acceptance of invasive screening, but may also have the potential to prevent cancer.

Based on the presence of certain genetic, epigenetic and related changes in the proteome of CRCs it has been tempting to assess the diagnostic accuracy of the presence of these changes in blood and stool for the purpose of CRC screening [5]–[7], [10], [11]. Until recently molecular colorectal testing has been based on the detection of genetic changes in stool. However, fecal DNA testing is expensive and the limited performance of these tests do not allow wide spread use as a molecular CRC screening tool [3].

Another non-invasive approach is the detection of epigenetic changes in blood and/or stool of patients with CRC (Tables S3 and S4). Several groups have reported a high frequency of methylated genes in CRC and in the stool of patients with CRC. Recently, we reported the identification of *SEPT9* and *ALX4* gene methylation as potential markers for CRCs [12]–[14]. Using a cut-off of 41.4 pg/ml, sensitivity and specificity of *ALX4* methylation in plasma was 83.3% and 70%, respectively [12]. In addition, recent analysis of *SEPT9* methylation in plasma revealed a sensitivity of 70% and specificity of 90% for the detection of patients with CRC [13], [14]. Based on these promising data we aimed to assess the potential role of these markers, both as single markers and as a panel, for the detection of colorectal precancerous lesions, i.e. for colorectal adenomas.

Similar to our previous reports on *ALX4* and *SEPT9* methylation in CRC patients [12]–[14], we found a high frequency of DNA methylation in plasma of patients with colorectal adenomas. Thus, we observed a significantly higher frequency of *ALX4* and *SEPT9* methylated DNA in plasma from patients with polyps versus healthy controls. In addition, the combined analysis of the two markers proved to be highly significant in the detection of colorectal polyps with a sensitivity and specificity reaching 71% and 95%, respectively, for the detection of advanced precancerous colorectal lesions. Thus, the performance of this marker panel was further enhanced after the detailed analysis of the histomorphological nature of the lesions that were observed in the patients with polyps. Accordingly, the combination of the two markers proved to be highly sensitive in the detection of advanced adenomas, which are defined clinically and pathologically as lesions that are ≥10 mm in size and exhibit features of potential malignant transformation (high grade intraepithelial neoplasia, villus component of adenoma, etc.).

Recently, other groups have also analysed the presence of methylation markers in body fluids from patients with CRCs and adenomas, with a special emphasis on molecular stool analysis. However, the sensitivity of single methylation markers does not exceed 57% and in blood almost none of these markers were found so far (Tables S3 and S4). Using qualitative methylation analysis based on realtime conditions we could detect methylated DNA in up to 71% of individuals with advanced adenomas. Our panel of markers is the first demonstration in blood of advanced precancerous colorectal lesion detection to show better performance than other marker panels tested in stool only (Tables S3 and S4).

We also analysed the methylation status by including other symptomatic patients groups with non-malignant intestinal diseases. Methylation of *SEPT9* was increased among high risk patients (67%) and patients with chronic inflammation (50%). Thus, our exploratory study indicates that SEPT9 may be able to identify potentially curable early cancers or even precancerous colorectal lesions in asymptomatic individuals. However, patients with concomitant inflammatory diseases or other increased cancer risk should be directed to colonoscopy according to current clinical routine without non-invasive first line screening [21], [22].

In conclusion, our study presents the first evidence for a novel approach for the detection of advanced precancerous colorectal lesions using methylation markers in plasma. Advanced precancerous colorectal lesions are associated with an increased frequency of methylated DNA in the plasma. Based on this study, the diagnosis of colorectal precancerous lesions may be possible with a non-invasive, sensitive and reliable plasma-based test using a panel of methylation markers. If confirmed in a larger patient population, a diagnostic test based on this panel could improve early detection of cancerous lesions by allowing preselection of patients with precancerous colorectal lesions that could be subsequently be removed by colonoscopy, thereby offering the potential to prevent colorectal cancer.

## Supporting Information

Figure S1 Performance comparison of HM/Methylight assays on tissue samples. 198 Colorectal cancer tissues and 22 normal colon mucosa samples were analyzed by quantitative real-time PCR. Performance for the HM/Methylight is demonstrated by ROC plot analysis.
(3.88 MB TIF)

Figure S2 Performance comparison of MSP/Methylight assays on tissue samples. 198 Colorectal cancer tissues and 22 normal colon mucosa samples were analyzed by quantitative real-time PCR. Performance for the MSP/Methylight is demonstrated by ROC plot analysis.
(3.83 MB TIF)

Table S1 Classification table: Marker panel for detection of advanced polyps.
(0.03 MB DOC)

Table S2 Summary of statistical results.
(0.04 MB DOC)

Table S3 Performance of methylation markers in stool and blood for detection of colorectal adenoma.
(0.04 MB DOC)

Table S4 Results of multipanel assays with methylation markers for diagnosis of colorectal cancer in stool.
(0.03 MB DOC)
