# Supplementary figures and images for: Performance of Epigenetic Markers SEPT9 and ALX4 in Plasma for Detection of Colorectal Precancerous Lesions
Source: PLoS One. 2010 Feb 4;5(2):e9061. doi: 10.1371/journal.pone.0009061 (PMC2816214; doi:10.1371/journal.pone.0009061)

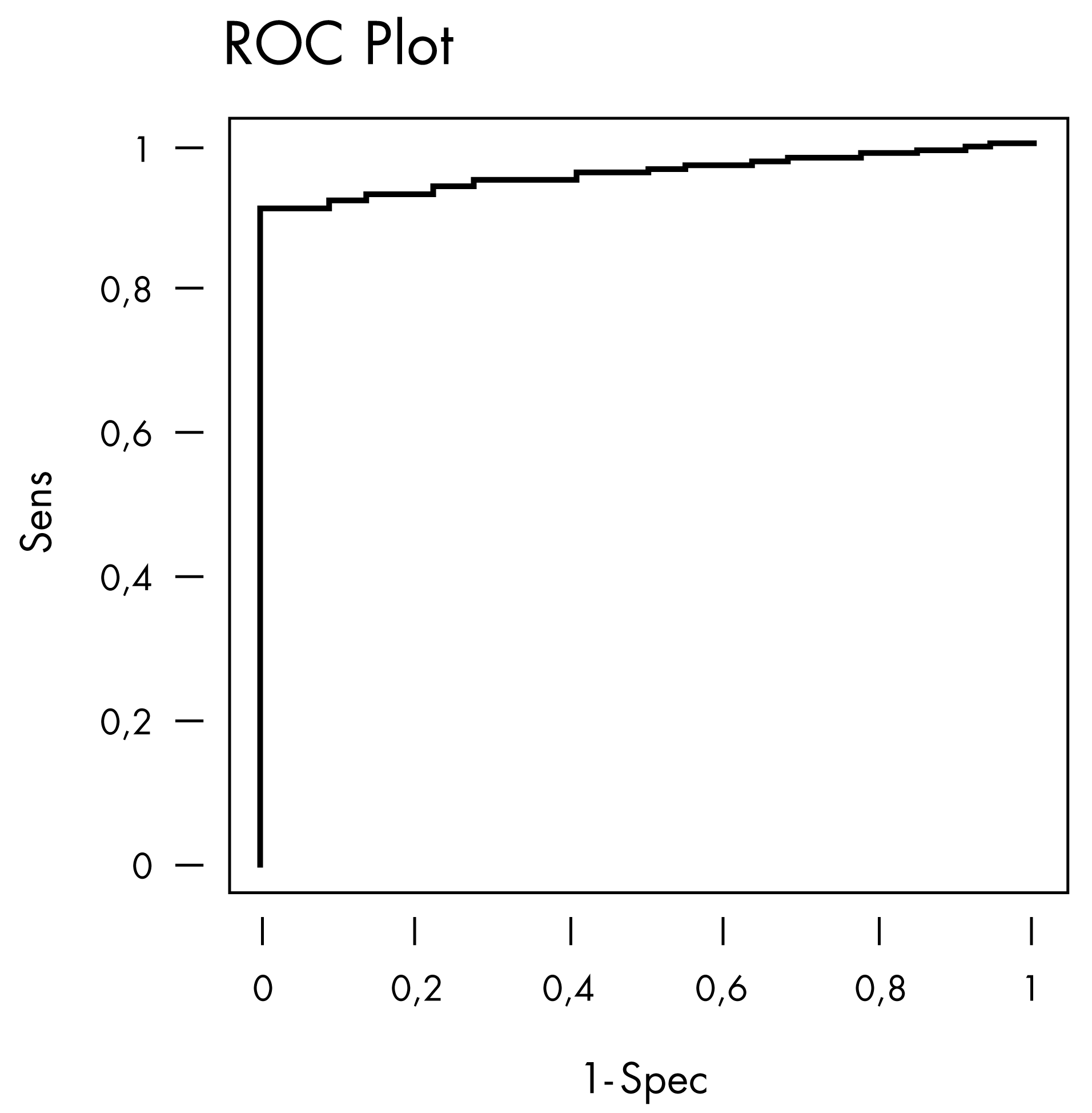

Supplement: Figure S1 — Performance comparison of HM/Methylight assays on tissue samples. 198 Colorectal cancer tissues and 22 normal colon mucosa samples were analyzed by quantitative real-time PCR. Performance for the HM/Methylight is demonstrated by ROC plot analysis. (3.88 MB TIF) [file pone.0009061.s001.tif]

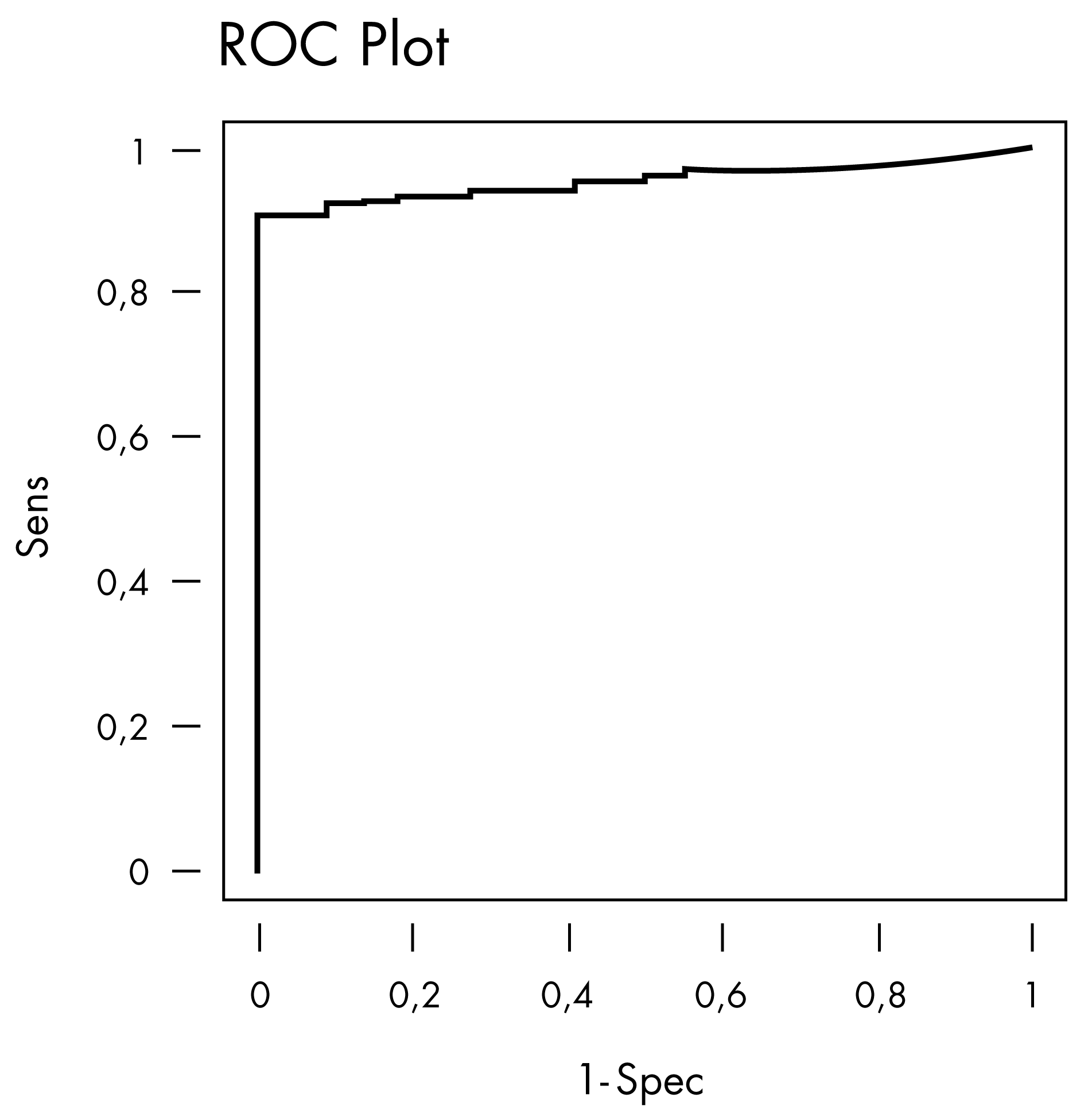

Supplement: Figure S2 — Performance comparison of MSP/Methylight assays on tissue samples. 198 Colorectal cancer tissues and 22 normal colon mucosa samples were analyzed by quantitative real-time PCR. Performance for the MSP/Methylight is demonstrated by ROC plot analysis. (3.83 MB TIF) [file pone.0009061.s002.tif]
