# Supplementary material for: Performance of Epigenetic Markers SEPT9 and ALX4 in Plasma for Detection of Colorectal Precancerous Lesions
Source: PLoS One. 2010 Feb 4;5(2):e9061. doi: 10.1371/journal.pone.0009061 (PMC2816214; doi:10.1371/journal.pone.0009061)
Supplement: Table S1 — Classification table: Marker panel for detection of advanced polyps. (0.03 MB DOC) [file pone.0009061.s003.doc]

Table S1. Classification table: Marker panel for detection of advanced polyps.

|  | Advanced Polyps  (tubular or tubulovillous, >10mm + aIEN**)** | |  |
| --- | --- | --- | --- |
|  | Absent | Present | Totals |
| Test Positive | 1 | 5 | 6 |
| Test Negative | 21 | 2 | 23 |
| Totals | 22 | 7 | 29 |
|  |  |  |  |
