# Supplementary material for: Performance of Epigenetic Markers SEPT9 and ALX4 in Plasma for Detection of Colorectal Precancerous Lesions
Source: PLoS One. 2010 Feb 4;5(2):e9061. doi: 10.1371/journal.pone.0009061 (PMC2816214; doi:10.1371/journal.pone.0009061)
Supplement: Table S2 — Summary of statistical results. (0.04 MB DOC) [file pone.0009061.s004.doc]

Table S2. Summary of statistical results.

|  |  | 95% Confidence Interval | |
| --- | --- | --- | --- |
|  | Estimated  Value | Lower Limit | Upper Limit |
| Prevalence | 0.24 | 0.11 | 0.44 |
| Sensitivity | 0.71 | 0.30 | 0.95 |
| Specificity | 0.95 | 0.75 | >0.99 |
| For any particular test result, the probability that it will be: | | | |
| Positive | 0.21 | 0.09 | 0.40 |
| Negative | 0.79 | 0.60 | 0.91 |
| For any particular positive test result, the probability that it is: | | |  |
| True Positive | 0.83 | 0.36 | 0.99 |
| False Positive | 0.17 | 0.01 | 0.64 |
| For any particular negative test result, the probability that it is: | | |  |
| True Negative | 0.91 | 0.70 | 0.98 |
| False Negative | 0.09 | 0.02 | 0.30 |
| likelihood Ratios:    [C] = conventional    [W] = weighted by prevalence | | |  |
| Positive [C] | 15.7 | 2.2 | 112.8 |
| Negative [C] | 0.30 | 0.09 | 0.97 |
| Positive [W] | 5 | 0.81 | 31.0 |
| Negative [W] | 0.10 | 0.03 | 0.36 |

(adapted from http://faculty.vassar.edu/lowry/clin1.html)
