# Supplementary material for: Performance of Epigenetic Markers SEPT9 and ALX4 in Plasma for Detection of Colorectal Precancerous Lesions
Source: PLoS One. 2010 Feb 4;5(2):e9061. doi: 10.1371/journal.pone.0009061 (PMC2816214; doi:10.1371/journal.pone.0009061)
Supplement: Table S3 — Performance of methylation markers in stool and blood for detection of colorectal adenoma. (0.04 MB DOC) [file pone.0009061.s005.doc]

Table S3. Performance of methylation markers in stool and blood for detection of colorectal adenoma.

| **Source** | **Gene** | **Methylation adenoma** | **Methylation in advanced adenoma (HIEN, >10mm)** | **Publication** |
| --- | --- | --- | --- | --- |
| **Stool** | **SFRP2** | **11/21 (52.4%)** | **7/10 (70%)** | **Huang ZH, World J Gastro 2007** |
|  | **HPP1/TPEF** | **12/21 (57%)** | **7/10 (70%)** |  |
|  | **MGMT** | **6/21 (28.6%)** | **4/10 (40%)** |  |
|  |  | **14/28 (50%)** |  | **Petko Z, Clin Cancer Res 2005** |
|  | **CDKN2** | **9/27 (33%)** |  |  |
|  | **HIC1** |  | **4/13 (31%)** | **Lenhard K, Clin Gastro Hep 2005** |
|  | **TFIP2** | **54/56 (97%)** |  | **Glöckner SC,**  **Cancer Res**  **2009** |
|  | **SFRP2** | **6/13 46%** |  | **Oberwalder M**  **Int J Colorectal Dis 2008** |
| **Blood** | **p16** | **0/34** |  | **Zou HZ, Clin Cancer Res 2002** |
|  | **HPP1/TPEF** | **1/8** |  | **Sabbioni S, Mol Diagn 2003** |
