# Supplementary material for: Performance of Epigenetic Markers SEPT9 and ALX4 in Plasma for Detection of Colorectal Precancerous Lesions
Source: PLoS One. 2010 Feb 4;5(2):e9061. doi: 10.1371/journal.pone.0009061 (PMC2816214; doi:10.1371/journal.pone.0009061)
Supplement: Table S4 — Results of multipanel assays with methylation markers for diagnosis of colorectal cancer in stool. (0.03 MB DOC) [file pone.0009061.s006.doc]

Table S4. Results of multipanel assays with methylation markers for diagnosis of colorectal cancer in stool.

| **Multipanel Assay (Genes)** | **Sensitivity** | **Specificity** | **Publication** |
| --- | --- | --- | --- |
| **SFRP2, HPP1, MGMT** | **80%** | **83.7%** | **Huang ZH, World J Gastro 2007** |
| **APC, ATM, hMLH1, sFRP2, HLTF, MGMT, GSTP1** | **68%** | **90%** | **Leung WK, Am J Gastro 2007** |
| **MGMT, CDKN2** | **55%** | **63%** | **Petko Z, Clin Cancer Res 2005** |
